# Construction of the circRNA-miRNA-mRNA axis based on ferroptosis-related gene AKR1C1 to explore the potential pathogenesis of abdominal aortic aneurysm

**DOI:** 10.1097/MD.0000000000038749

**Published:** 2024-06-28

**Authors:** Xuehua Huang, Huanhuan Deng

**Affiliations:** a Department of Neonatology, the First Hospital of China Medical University, Shenyang, China; b Department of Nephrology, the First Hospital of China Medical University, Shenyang, China.

**Keywords:** abdominal aortic aneurysm, circRNA-miRNA-mRNA axis, ferroptosis, pathogenesis

## Abstract

Abdominal aortic aneurysm (AAA) is a cardiovascular disease that seriously threatens human health and brings huge economic burden. At present, its pathogenesis remains unclear and its treatment is limited to surgical treatment. With the deepening and analysis of studies on the mechanism of ferroptosis, a new idea has been provided for the clinical management of AAA patients, including diagnosis, treatment and prevention. Therefore, this paper aims to construct a competitive endogenous RNA (ceRNA) regulatory axis based on ferroptosis to preliminarily explore the pathogenesis and potential therapeutic targets of AAA. We obtained upregulated and downregulated ferroptosis-related DEGs (FRGs) from GSE144431 dataset and 60 known ferroptosis-related genes. Pearson correlation analysis was used to find aldoketone reductase 1C (AKR1C1) in AAA samples. Enrichment analysis of these genes was performed via Gene Ontology (GO) and Kyoto Encyclopedia of Genes and Genomes (KEGG). Correlation test between immune cells and AKR1C1 was investigated through single-sample gene set enrichment analysis (ssGSEA). The AKR1C1-miRNA pairs were predicted by the TargetScan database and miRWalk database. Circular RNA (CircRNA)-miRNA pairs were selected by the CircInteractome database. Overlapping miRNA between circRNA-miRNA and AKR1C1-miRNA pairs was visualized by Venn diagram. Finally, the circRNA-miRNA-mRNA axis was constructed by searching for upstream circRNA and downstream mRNA of overlapping miRNA. Only one downregulated AKR1C1 gene was found in GSE144431 and 60 ferroptosis-related genes. Functional Enrichment and Pathway Analysis of AKR1C1-related genes were further explored, and it was observed that they were mainly enriched in “response to oxidative stress,” “glutathione biosynthetic process” and “nonribosomal peptide biosynthetic process,” “Ferroptosis,” “Glutathione metabolism” and “Chemical carcinogenesis-reactive oxygen species.” They were also found to be significantly associated with most immune cells, including Activated Dendritic cells, CD56dim Natural killer cells, Gamma Delta T cells, Immature B cells, Plasmacytoid dendritic cell, Type 2 T helper cell, Activated CD4 T cell and Type 1 T helper cell. Has_circ_0005073-miRNA-543 and AKR1C1-miRNA-543 were identified by Online Database analysis. Therefore, we have established the has_circ_0005073/miRNA-543/AKR1C1 axis in AAA. We found AKR1C1 was differentially expressed between normal and AAA groups. Based on AKR1C1, we constructed the has_circ_0005073/miRNA-543/AKR1C1 axis to analyze AAA.

**Key Points**

1. Ferroptosis-related gene AKR1C1 played a vital role in pathogenesis of abdominal aortic aneurysm (AAA).
2. Has_circ_0005073/miRNA-543/AKR1C1 axis may be a potential regulatory network in the occurrence and development of AAA.

## 1. Introduction

Abdominal aortic aneurysm (AAA) is defined as the enlargement of the maximal diameter of the subrenal aorta to 1.5 times the diameter of the adjacent normal aorta or >30 mm.^[1]^ AAA is characterized by continuous expansion and weakening of the local abdominal aorta, which is more common in elderly men, and the onset is more insidious.^[2–4]^ Rupture is the most important and common fatal complication of AAA, and about 150,000–200,000 deaths worldwide are attributed to AAA rupture every year, with the overall mortality rate as high as 65% to 80%.^[5]^ Given AAA low detection and high rupture rates, reliable, available and low-cost biomarkers are needed. In addition, there is currently no effective treatment to limit the development of AAA and prevent AAA rupture, and the only strategy is to continuously monitor aneurysm size before surgery.^[4,6]^ Therefore, it is urgent to explore the pathogenesis of AAA to provide theoretical basis for prevention and treatment.

The pathogenesis of AAA is relatively complex, and multiple factors are involved, mainly including biomechanical changes,^[7]^ the formation of Lumen thrombosis,^[8]^ vascular smooth muscle cell (VSMC) phenotype transformation and apoptosis,^[9]^ extracellular matrix remodeling,^[10]^ the inflammatory cell infiltration,^[11]^ vascular aging.^[12]^ Although relevant research reports emerge endlessly, the specific causes of AAA are still not fully clarified.

Ferroptosis was first proposed by Brent R. Stockwell in 2012 as a unique form of cell death different from apoptosis, necrotic apoptosis and autophagy.^[13]^ The essence of ferroptosis is oxidative damage, mainly due to mitochondrial changes caused by excessive accumulation of iron-dependent lipid peroxidation products.^[14]^ A large number of studies have shown that ferroptosis plays an important role in the occurrence and development of cardiovascular diseases, such as atherosclerosis, heart failure, myocardial infarction and AAA.^[15]^ Studies have suggested that iron levels in aneurysm tissues are significantly increased, and oxidative stress and inflammatory response induced by iron overload aggravate the progression of AAA.^[16]^ Sampilvanjil et al found that cigarette smoke extract can induce ferroptosis in VSMCs.^[17]^ Therefore, we further explore how ferroptosis specifically affects the formation and progression of AAA.

With the deepening of research, epigenetic modifications including histone DNA methylation, protein modification and non-coding RNA (ncRNA) modification are considered to be important contributors to AAA.^[18–21]^ NcRNA is composed of a variety of RNA transcripts, including microRNA (miRNA), long non-coding RNA (lncRNA) and circular RNA (circRNA).^[22]^ CircRNA and lncRNA can act as competitive endogenous RNAs (ceRNAs) and sponge miRNAs, thereby counteracting the negative regulation of miRNAs on target mRNAs.^[23]^ Yue et al revealed that circCBFB/miR-28-5p/GRIA4/LYPD3 axis regulated apoptosis of VSMC and participated in the formation of AAA.^[24]^ Yang et al proposed that circRNACCDC66/miR-342-3p/CCDC6 axis mediated VSMC proliferation to promote the occurrence and development of AAA.^[25]^ Therefore, circRNA-miRNA-mRNA axis may play an important role in the pathogenesis of AAA.

In our study, differentially expressed genes (DEGs) and circRNAs (DECs) related to ferroptosis were analyzed based on GEO database. CircInteractome database was used to predict circRNA-miRNA pairs and TargetScan and miRWalk database was performed to predict mRNA-miRNA pairs to find overlapping miRNAs. Based on the above premises, we carried on the preliminary exploration to the pathogenesis of AAA.

## 2. Materials and methods

### 2.1. Data extraction

Microarray data of AAA patients were downloaded from GEO database (https://www.ncbi.nlm.nih.gov/geo/). GSE47472, conducted by GPL10558 (Illumina HumanHT-12 V4.0 expression beadchip), included mRNA data of 8 normal samples and 14 AAA samples. GSE144431, conducted by GPL21825 (074301 Arraystar Human ncRNA microarray V2), included circRNA data of 4 normal samples and 4 AAA samples. GSE236869 was used as a validation dataset. 60 ferroptosis-related genes were retrieved from our previously published study.^[26]^

### 2.2. Differential expression analysis

DEGs and differentially expressed DECs were ascertained by performing the limma R package with the statistical threshold of |log2fold change (FC)| > 0.7 and *P* < .05 and |log2fold change (FC)| > 1.5 and *P* < .01 in R studio (version 4.1.1), respectively. The DEGs and DECs were divided into the upregulated and downregulated groups. Subsequently, these genes were visualized by Volcano plot and Heatmap. Meanwhile, Venn diagram was utilized to show ferroptosis-related DEGs (FRGs) between upregulated or downregulated DEGs and ferroptosis-related genes.

### 2.3. Correlation analysis

Pearson correlation test was used to screen the co-expressed genes with the statistical threshold of |R| > 0.7 and *P* < .05 in R package. Single-sample gene set enrichment analysis (ssGSEA) was applied to evaluate the proportion of 28 immune cells in normal and AAA groups and the degree of correlation between aldoketone reductase 1C (AKR1C1) and 28 immune cells with same threshold as above.

### 2.4. Functional enrichment and pathway analysis

To elaborate the main functions and pathways of co-expressed genes, the Database for Annotation, Visualization and Integrated Discovery online tool (version 6.8) (https://david.ncifcrf.gov/) was chosen for the Gene Ontology (GO) and Kyoto Encyclopedia of Genes and Genomes (KEGG) pathway enrichment analyses.

### 2.5. Construction of circRNA-miRNA-mRNA axis

The CircInteractome database (version 1.0) (https://circinteractome.nia.nih.gov/index.html) was used to predict circRNA-miRNA pairs. The mRNA-miRNA pairs were predicted by the TargetScan database (version 7.2) (http://www.targetscan.org) and miRWalk database (version 2.0) (http://mirwalk.umm.uni-heidelberg.de/). Overlapping miRNA between circRNA-miRNA and mRNA-miRNA pairs was illustrated by Venn diagram. Therefore, the circRNA-miRNA-mRNA axis was constructed by searching for upstream circRNA and downstream mRNA of overlapping miRNA.

### 2.6. Statistical analysis

Mann-Whitney test was conducted to compare data between different groups. Statistical analysis and data visualization were performed by using R Studio (V.4.1.1), GraphPad Prism (V.8.0) and AutoDock (V.4.2) software. *P* < .05 was considered statistically significant.

### 2.7. Ethics statement

The data for this study comes from public databases, does not involve the testing of human and animal samples. Therefore, ethical approval is not applicable to this study.

## 3. Results

### 3.1. Identification of candidate FRG

**Figure 1.**
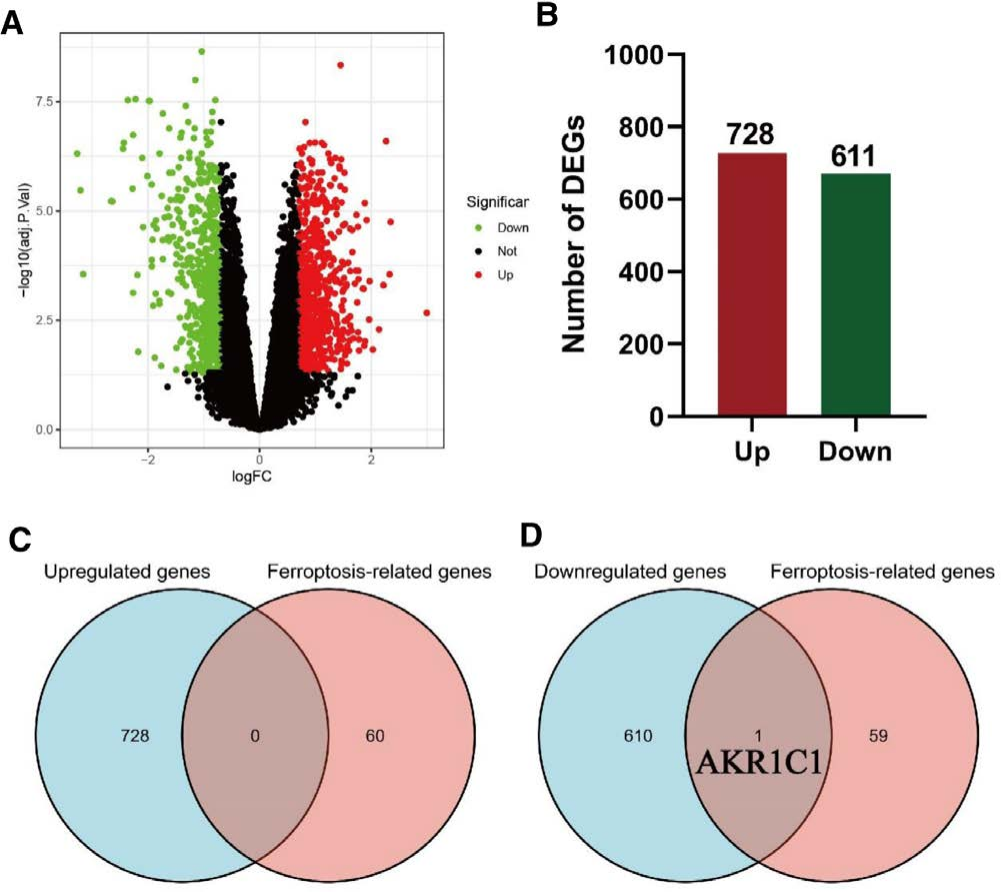
AKR1C1 was identified as the key ferroptosis-related differentially expressed gene (FRG). (A) Volcano Plot shows DEGs from GSE144431 dataset. (B) The numbers of upregulated and downregulated DEGs. (C-D) Venn Diagram shows the intersection of upregulated (C) and downregulated (D) DEGs with senescence-related genes. AKR1C = aldoketone reductase 1C, DEGs = differentially expressed genes.

Based on the GSE47472 dataset in the GEO database, DEGs were analyzed between the AAA group and the control group, and the results as shown in the Volcano map (Fig. 1A), contained 728 upregulated genes and 611 downregulated genes (Fig. 1B). Subsequently, we intersected them with ferroptosis-related genes respectively. Venn diagram showed that no overlapping genes were found in the upregulated genes (Fig. 1C), but AKR1C1 was identified as FR-DEG in the downregulated genes (Fig. 1D).

### 3.2. The potential biological functions of AKR1C1

**Figure 2.**
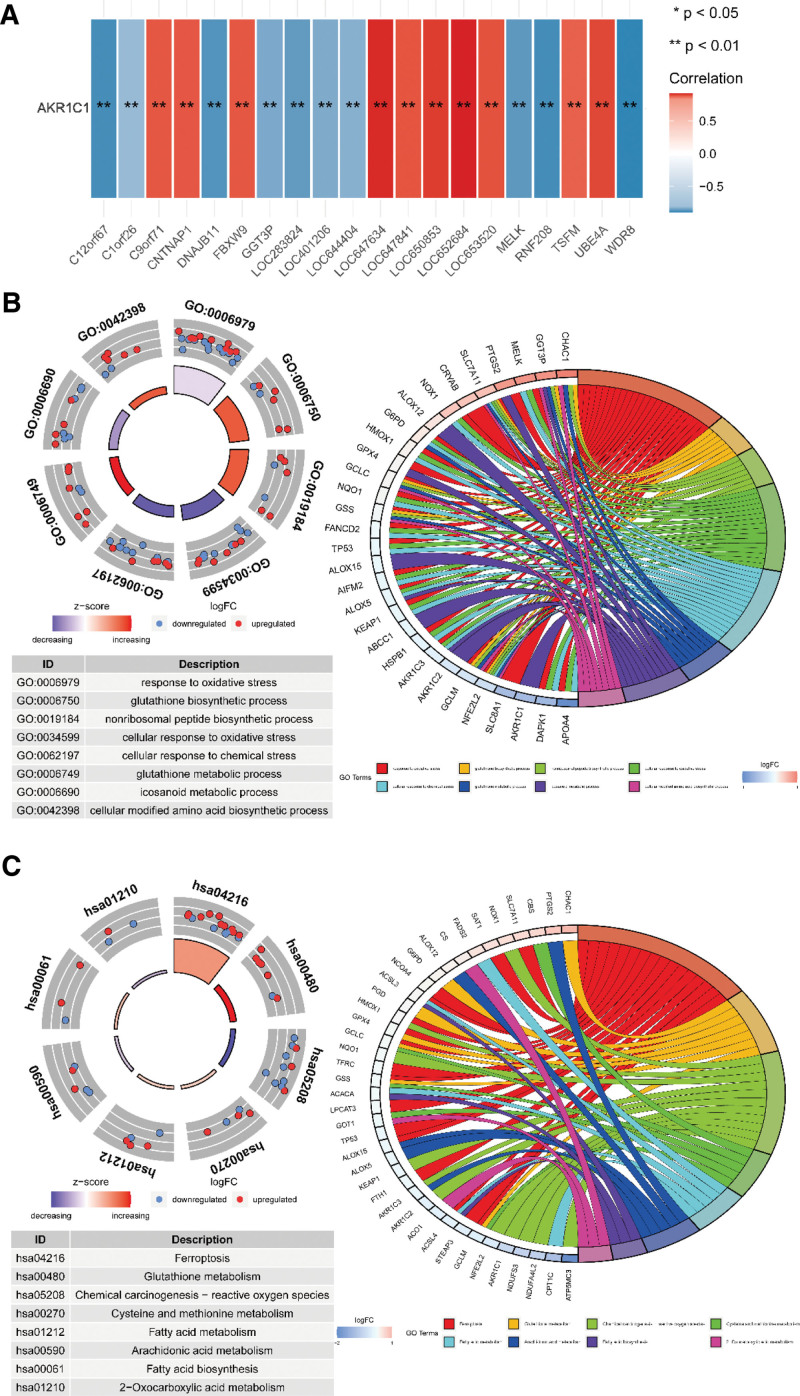
The potential biological functions of AKR1C1. (A) The top 10 genes of positive and negative correlated with AKR1C1. (B-C) GO (B) and KEGG (C) enrichment analysis for these genes correlated with AKR1C1. AKR1C = aldoketone reductase 1C, GO = Gene Ontology, KEGG = Kyoto encyclopedia of genes and genomes.

Based on the above results, we explored the biological functions of AKR1C1. Co-expression analysis found that 381 genes were positively correlated with AKR1C1, and 111 genes were negatively correlated with AKR1C1. The top 10 genes with positive and negative correlations were drawn in the below figure (Fig. 2A). At the same time, we further explore these genes related AKR1C1 through GO term and KEGG analyses. GO enrichment analysis revealed that they were mainly involved in “response to oxidative stress,” “glutathione biosynthetic process” and “nonribosomal peptide biosynthetic process” (Fig. 2B). KEGG enrichment analysis found that they were mainly enriched in “Ferroptosis,” “Glutathione metabolism” and “Chemical carcinogenesis-reactive oxygen species” (Fig. 2C).

### 3.3. The roles of AKR1C1 in the immune microenvironment

**Figure 3.**
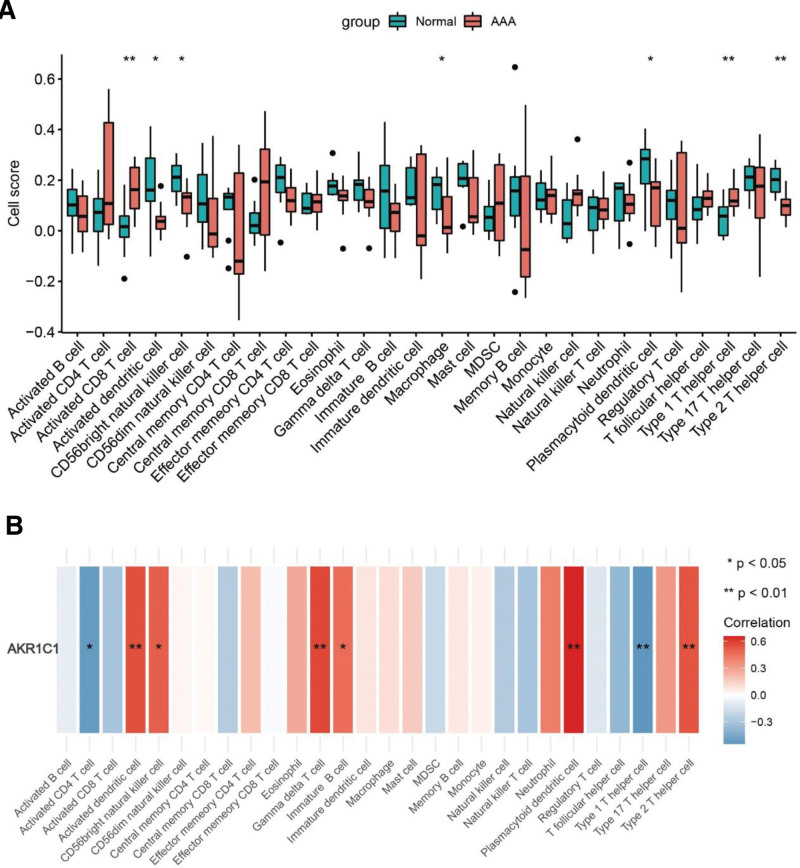
AKR1C1 is closely related with immune infiltration. (A) The results display of the expression levels of 28 immune cells between the normal group and AAA group. (B) The correlation of AKR1C1 and 28 immune cells in AAA group. *P < .05, ** *P* < .01. AAA = abdominal aortic aneurysm, AKR1C = aldoketone reductase 1C.

We further analyzed the role of immune cell infiltration and how AKR1C1 plays a regulatory role in the pathogenesis of AAA. Firstly, we detected the differential expression of 28 immune cells between AAA group and control group by ssGSEA. The results showed that the Activated CD8 T cell and Type 1 T helper cell were highly expressed in AAA group, the expression levels of Activated dendritic cell, CD56dim natural killer cell, Macrophage cell, Plasmacytoid dendritic cell and Type 2 T helper cell were low in AAA group compared with control group (Fig. 3A). In addition, we observed the correlation degree of AKR1C1 and 28 immune cells. The results showed that the Activated dendritic cell, CD56dim natural killer cell, Gamma delta T cell, Immature B cell, Plasmacytoid dendritic cell and Type 2 T helper cell were positively correlated, while Activated CD4 T cell and Type 1 T helper cell were negatively correlated (Fig. 3B). Therefore, we believe that AKR1C1 may regulate the Activated dendritic cell, CD56dim natural killer cell, and Plasmacytoid dendritic cell, particularly Type 1 T helper cell and Type 2 T helper cell, to participate in the occurrence and development of AAA.

### 3.4. Construction of circRNA-miRNA-mRNA axis

**Figure 4.**
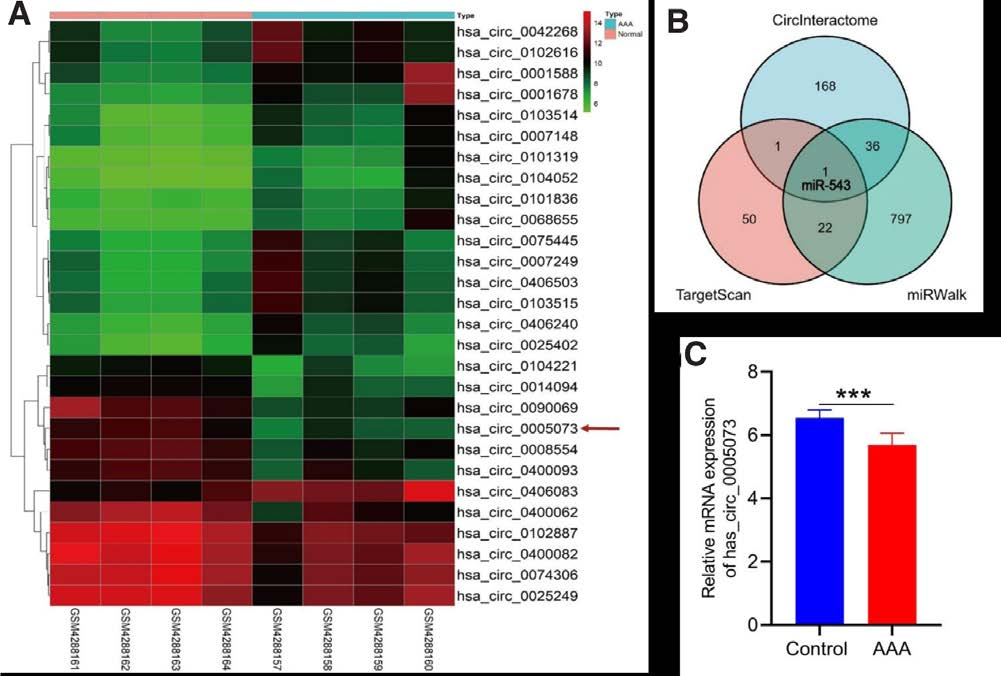
(A) Heatmap shows DECs from GSE144431 dataset. (B) The intersection of CircInteractome, TargetScan and miRWalk database. (C) The expression level of Has circ 0005073 in the validation dataset. DECs = DEGs and circRNAs.

On account of AKR1C1 and immune infiltration being closely related, we further searched for AKR1C1 possible upstream regulatory factors. At first, we analyzed the DECs between the AAA group and the control group in the GSE144431 dataset, and drew a Heatmap to demonstrate the upregulated and downregulated DECs (Fig. 4A). Since AKR1C1 was a downregulated gene in the AAA group, we selected downregulated DECs and predicted the miRNAs that these DECs might bind by CircInteractome database (Table 1). Analogously, we predicted the miRNAs that AKR1C1 might bind by TargetScan database and miRWalk Database (Table 2). Venn diagram was used to find common miRNA (miRNA-543) in all 3 of them, and then has_circ_0005073 was reversely searched according to miRNA-543 (Fig. 4B). The results of validation set showed that the expression of has_circ_0005073 was significantly reduced in AAA patients (Fig. 4C). Thus, we preliminarily believe that the has_circ_0005073/miRNA-543/AKR1C1 axis may be involved in the pathogenesis of AAA.

**Table 1:** Potential miRNAs regulating differential genes in CircInteractome database.

hsa-miR-1179
hsa-miR-1183
hsa-miR-1184
hsa-miR-1200
hsa-miR-1224-3p
hsa-miR-1228
hsa-miR-1231
hsa-miR-1236
hsa-miR-1238
hsa-miR-1243
hsa-miR-1245
hsa-miR-1246
hsa-miR-1248
hsa-miR-1251
hsa-miR-1253
hsa-miR-1257
hsa-miR-1261
hsa-miR-1270
hsa-miR-127-5p
hsa-miR-1276
hsa-miR-1278
hsa-miR-1283
hsa-miR-1290
hsa-miR-1292
hsa-miR-1294
hsa-miR-1299
hsa-miR-1307
hsa-miR-1324
hsa-miR-136
hsa-miR-140-3p
hsa-miR-142-5p
hsa-miR-149
hsa-miR-155
hsa-miR-182
hsa-miR-1827
hsa-miR-183
hsa-miR-186
hsa-miR-194
hsa-miR-197
hsa-miR-217
hsa-miR-223
hsa-miR-224
hsa-miR-323-3p
hsa-miR-324-5p
hsa-miR-326
hsa-miR-330-5p
hsa-miR-331-5p
hsa-miR-335
hsa-miR-337-3p
hsa-miR-338-5p
hsa-miR-382
hsa-miR-383
hsa-miR-384
hsa-miR-411
hsa-miR-421
hsa-miR-450b-3p
hsa-miR-485-3p
hsa-miR-487a
hsa-miR-488
hsa-miR-490-5p
hsa-miR-494
hsa-miR-495
hsa-miR-498
hsa-miR-502-5p
hsa-miR-507
hsa-miR-510
hsa-miR-512-5p
hsa-miR-513a-3p
hsa-miR-513a-5p
hsa-miR-515-3p
hsa-miR-515-5p
hsa-miR-516b
hsa-miR-518a-5p
hsa-miR-519e
hsa-miR-520f
hsa-miR-520g
hsa-miR-520h
hsa-miR-527
hsa-miR-543
hsa-miR-548b-3p
hsa-miR-548c-3p
hsa-miR-548g
hsa-miR-548k
hsa-miR-548l
hsa-miR-548m
hsa-miR-548p
hsa-miR-555
hsa-miR-556-3p
hsa-miR-556-5p
hsa-miR-557
hsa-miR-558
hsa-miR-561
hsa-miR-562
hsa-miR-567
hsa-miR-570
hsa-miR-571
hsa-miR-576-5p
hsa-miR-578
hsa-miR-580
hsa-miR-581
hsa-miR-582-3p
hsa-miR-583
hsa-miR-584
hsa-miR-586
hsa-miR-587
hsa-miR-593
hsa-miR-600
hsa-miR-604
hsa-miR-605
hsa-miR-606
hsa-miR-607
hsa-miR-609
hsa-miR-610
hsa-miR-614
hsa-miR-616
hsa-miR-619
hsa-miR-620
hsa-miR-622
hsa-miR-623
hsa-miR-624
hsa-miR-625
hsa-miR-628-3p
hsa-miR-630
hsa-miR-635
hsa-miR-636
hsa-miR-640
hsa-miR-643
hsa-miR-646
hsa-miR-647
hsa-miR-649
hsa-miR-656
hsa-miR-661
hsa-miR-662
hsa-miR-766
hsa-miR-769-3p
hsa-miR-769-5p
hsa-miR-873
hsa-miR-876-3p
hsa-miR-889
hsa-miR-890
hsa-miR-891a
hsa-miR-892a
hsa-miR-936
hsa-miR-938
hsa-miR-940
hsa-miR-942
hsa-miR-1206
hsa-miR-1248
hsa-miR-1278
hsa-miR-1299
hsa-miR-324-5p
hsa-miR-526b
hsa-miR-548k
hsa-miR-548l
hsa-miR-579
hsa-miR-767-3p
hsa-miR-889
hsa-miR-1203
hsa-miR-1288
hsa-miR-183
hsa-miR-326
hsa-miR-330-5p
hsa-miR-331-5p
hsa-miR-508-3p
hsa-miR-520f
hsa-miR-548b-3p
hsa-miR-548p
hsa-miR-561
hsa-miR-580
hsa-miR-589
hsa-miR-621
hsa-miR-622
hsa-miR-628-3p
hsa-miR-636
hsa-miR-653
hsa-miR-338-3p
hsa-miR-570
hsa-miR-629
hsa-miR-766
hsa-miR-891b
hsa-miR-1179
hsa-miR-1182
hsa-miR-1205
hsa-miR-1208
hsa-miR-1227
hsa-miR-1228
hsa-miR-1229
hsa-miR-1231
hsa-miR-1236
hsa-miR-1248
hsa-miR-1249
hsa-miR-1251
hsa-miR-1252
hsa-miR-1253
hsa-miR-1256
hsa-miR-1264
hsa-miR-127-5p
hsa-miR-1279
hsa-miR-1281
hsa-miR-1286
hsa-miR-1294
hsa-miR-1295
hsa-miR-1299
hsa-miR-1304
hsa-miR-1305
hsa-miR-136
hsa-miR-140-3p
hsa-miR-145
hsa-miR-146b-3p
hsa-miR-149
hsa-miR-1825
hsa-miR-1827
hsa-miR-188-3p
hsa-miR-192
hsa-miR-194
hsa-miR-197
hsa-miR-198
hsa-miR-215
hsa-miR-323-3p
hsa-miR-324-5p
hsa-miR-326
hsa-miR-330-3p
hsa-miR-330-5p
hsa-miR-331-3p
hsa-miR-338-3p
hsa-miR-370
hsa-miR-377
hsa-miR-384
hsa-miR-421
hsa-miR-432
hsa-miR-450b-3p
hsa-miR-485-3p
hsa-miR-486-3p
hsa-miR-488
hsa-miR-495
hsa-miR-502-5p
hsa-miR-507
hsa-miR-512-5p
hsa-miR-513a-5p
hsa-miR-515-5p
hsa-miR-516b
hsa-miR-518a-5p
hsa-miR-526b
hsa-miR-527
hsa-miR-545
hsa-miR-548l
hsa-miR-548m
hsa-miR-548p
hsa-miR-549
hsa-miR-555
hsa-miR-557
hsa-miR-558
hsa-miR-567
hsa-miR-571
hsa-miR-576-3p
hsa-miR-582-3p
hsa-miR-583
hsa-miR-598
hsa-miR-600
hsa-miR-605
hsa-miR-615-5p
hsa-miR-618
hsa-miR-621
hsa-miR-622
hsa-miR-625
hsa-miR-626
hsa-miR-634
hsa-miR-635
hsa-miR-637
hsa-miR-640
hsa-miR-646
hsa-miR-648
hsa-miR-649
hsa-miR-661
hsa-miR-665
hsa-miR-767-5p
hsa-miR-769-3p
hsa-miR-873
hsa-miR-874
hsa-miR-885-3p
hsa-miR-936
hsa-miR-938
hsa-miR-941
hsa-miR-942
hsa-miR-324-5p
hsa-miR-338-3p
hsa-miR-616
hsa-miR-766
hsa-miR-1303
hsa-miR-1825
hsa-miR-331-3p
hsa-miR-346
hsa-miR-384
hsa-miR-515-5p
hsa-miR-558
hsa-miR-659
hsa-miR-766
hsa-miR-877

miRNA = microRNA.

**Table 2:** Potential miRNAs binding to AKR1C1 in TargetScan database and miRWalk database.

Database	TargetScan	miRWalk
1	hsa-miR-6810-5p	hsa-miR-1228-3p
2	hsa-miR-3191-3p	hsa-miR-6775-5p
3	hsa-miR-4652-5p	hsa-miR-17-3p
4	hsa-miR-3144-5p	hsa-miR-204-5p
5	hsa-miR-450a-2-3p	hsa-miR-106b-3p
6	hsa-miR-6857-5p	hsa-miR-99b-3p
7	hsa-miR-2110	hsa-miR-412-3p
8	hsa-miR-3150a-3p	hsa-miR-511-5p
9	hsa-miR-6763-5p	hsa-miR-671-5p
10	hsa-miR-5572	hsa-miR-1229-3p
11	hsa-miR-4260	hsa-miR-1303
12	hsa-miR-543	hsa-miR-1288-3p
13	hsa-miR-3143	hsa-miR-718
14	hsa-miR-194-5p	hsa-miR-4279
15	hsa-miR-6867-5p	hsa-miR-4429
16	hsa-miR-203a-3p.1	hsa-miR-4447
17	hsa-miR-203a-3p.2	hsa-miR-6075
18	hsa-miR-4728-3p	hsa-miR-6133
19	hsa-miR-155-5p	hsa-miR-6514-5p
20	hsa-miR-4280	hsa-miR-6766-3p
21	hsa-miR-548t-5p	hsa-miR-6766-3p
22	hsa-miR-548az-5p	hsa-miR-6819-3p
23	hsa-miR-6124	hsa-miR-6826-5p
24	hsa-miR-4668-5p	hsa-miR-6859-3p
25	hsa-miR-3153	hsa-miR-6869-5p
26	hsa-miR-6733-5p	hsa-miR-6890-3p
27	hsa-miR-6739-5p	hsa-miR-7154-3p
28	hsa-miR-4646-5p	hsa-miR-7843-3p
29	hsa-miR-204-3p	hsa-miR-8085
30	hsa-miR-4699-3p	hsa-miR-9898
31	hsa-miR-32-3p	hsa-miR-5588-5p
32	hsa-miR-380-3p	hsa-miR-557
33	hsa-miR-3613-3p	hsa-miR-4448
34	hsa-miR-3163	hsa-miR-6787-5p
35	hsa-miR-4803	hsa-miR-11400
36	hsa-miR-4522	hsa-miR-18a-5p
37	hsa-miR-181b-2-3p	hsa-let-7g-5p
38	hsa-miR-181b-3p	hsa-miR-1289
39	hsa-miR-4420	hsa-miR-1976
40	hsa-miR-653-3p	hsa-miR-3615
41	hsa-miR-6837-3p	hsa-miR-4469
42	hsa-miR-412-3p	hsa-miR-4676-3p
43	hsa-miR-6754-3p	hsa-miR-4690-5p
44	hsa-miR-7161-3p	hsa-miR-5706
45	hsa-miR-3935	hsa-miR-5739
46	hsa-miR-376a-2-5p	hsa-miR-6070
47	hsa-miR-1183	hsa-miR-6127
48	hsa-miR-181a-2-3p	hsa-miR-6774-3p
49	hsa-miR-6509-3p	hsa-miR-6803-5p
50	hsa-miR-1273g-3p	hsa-miR-11181-3p
51	hsa-miR-4436b-5p	hsa-miR-5093
52	hsa-miR-3692-3p	hsa-miR-205-3p
53	hsa-miR-3924	hsa-miR-1234-3p
54	hsa-miR-5580-3p	hsa-miR-3141
55	hsa-let-7f-1-3p	hsa-miR-6790-3p
56	hsa-let-7a-3p	hsa-miR-6827-3p
57	hsa-miR-98-3p	hsa-miR-6769b-5p
58	hsa-let-7b-3p	hsa-miR-4433b-3p
59	hsa-miR-1185-1-3p	hsa-miR-3911
60	hsa-let-7f-2-3p	hsa-miR-515-3p
61	hsa-miR-1185-2-3p	hsa-let-7a-5p
62	hsa-miR-4789-5p	hsa-let-7a-2-3p
63	hsa-miR-590-3p	hsa-let-7b-3p
64	hsa-miR-4775	hsa-let-7c-3p
65	hsa-miR-4735-5p	hsa-let-7d-5p
66	hsa-miR-3144-3p	hsa-miR-15a-3p
67	hsa-miR-4528	hsa-miR-18a-3p
68	hsa-miR-382-3p	hsa-miR-24-1-5p
69	hsa-miR-6506-3p	hsa-miR-28-3p
70	hsa-miR-3065-3p	hsa-miR-29a-3p
71	hsa-miR-4477a	hsa-miR-31-5p
72	hsa-miR-3140-3p	hsa-miR-92a-3p
73	hsa-miR-514b-5p	hsa-miR-92a-2-5p
74	hsa-miR-513c-5p	hsa-miR-92a-2-5p
75		hsa-miR-93-5p
76		hsa-miR-93-5p
77		hsa-miR-96-3p
78		hsa-miR-99a-5p
79		hsa-miR-101-5p
80		hsa-miR-29b-2-5p
81		hsa-miR-103a-3p
82		hsa-miR-103a-1-5p
83		hsa-miR-105-5p
84		hsa-miR-197-3p
85		hsa-miR-198
86		hsa-miR-198
87		hsa-miR-199a-5p
88		hsa-miR-208a-5p
89		hsa-miR-208a-5p
90		hsa-miR-129-1-3p
91		hsa-miR-147a
92		hsa-miR-34a-3p
93		hsa-miR-199b-5p
94		hsa-miR-199b-5p
95		hsa-miR-210-3p
96		hsa-miR-211-5p
97		hsa-miR-212-5p
98		hsa-miR-214-5p
99		hsa-miR-215-3p
100		hsa-miR-216a-3p
101		hsa-miR-222-5p
102		hsa-miR-222-3p
103		hsa-miR-224-5p
104		hsa-let-7g-3p
105		hsa-miR-23b-5p
106		hsa-miR-27b-5p
107		hsa-miR-30b-3p
108		hsa-miR-122-5p
109		hsa-miR-125b-1-3p
110		hsa-miR-128-1-5p
111		hsa-miR-132-5p
112		hsa-miR-132-3p
113		hsa-miR-135a-3p
114		hsa-miR-140-3p
115		hsa-miR-145-5p
116		hsa-miR-191-3p
117		hsa-miR-9-5p
118		hsa-miR-125a-5p
119		hsa-miR-125a-3p
120		hsa-miR-129-2-3p
121		hsa-miR-134-3p
122		hsa-miR-134-3p
123		hsa-miR-149-5p
124		hsa-miR-149-3p
125		hsa-miR-149-3p
126		hsa-miR-154-5p
127		hsa-miR-188-5p
128		hsa-miR-188-3p
129		hsa-miR-320a-5p
130		hsa-miR-320a-3p
131		hsa-miR-200c-5p
132		hsa-miR-155-5p
133		hsa-miR-194-3p
134		hsa-miR-29c-5p
135		hsa-miR-101-2-5p
136		hsa-miR-219a-2-3p
137		hsa-miR-301a-5p
138		hsa-miR-296-5p
139		hsa-miR-296-5p
140		hsa-miR-296-3p
141		hsa-miR-130b-5p
142		hsa-miR-361-3p
143		hsa-miR-302b-5p
144		hsa-miR-302c-3p
145		hsa-miR-302d-5p
146		hsa-miR-302d-3p
147		hsa-miR-376a-5p
148		hsa-miR-376a-5p
149		hsa-miR-377-5p
150		hsa-miR-378a-5p
151		hsa-miR-330-5p
152		hsa-miR-330-5p
153		hsa-miR-337-3p
154		hsa-miR-135b-5p
155		hsa-miR-135b-3p
156		hsa-miR-148b-3p
157		hsa-miR-339-5p
158		hsa-miR-339-3p
159		hsa-miR-339-3p
160		hsa-miR-335-3p
161		hsa-miR-345-3p
162		hsa-miR-196b-3p
163		hsa-miR-196b-3p
164		hsa-miR-423-3p
165		hsa-miR-18b-5p
166		hsa-miR-20b-3p
167		hsa-miR-20b-3p
168		hsa-miR-449a
169		hsa-miR-431-5p
170		hsa-miR-433-5p
171		hsa-miR-452-5p
172		hsa-miR-483-3p
173		hsa-miR-484
174		hsa-miR-485-3p
175		hsa-miR-486-5p
176		hsa-miR-490-3p
177		hsa-miR-491-5p
178		hsa-miR-511-5p
179		hsa-miR-146b-3p
180		hsa-miR-498-5p
181		hsa-miR-519e-3p
182		hsa-miR-520f-3p
183		hsa-miR-525-5p
184		hsa-miR-525-3p
185		hsa-miR-523-3p
186		hsa-miR-520b-5p
187		hsa-miR-520b-3p
188		hsa-miR-520c-3p
189		hsa-miR-518c-5p
190		hsa-miR-518c-5p
191		hsa-miR-520g-3p
192		hsa-miR-516b-3p
193		hsa-miR-518e-3p
194		hsa-miR-518d-3p
195		hsa-miR-517c-3p
196		hsa-miR-516a-3p
197		hsa-miR-519a-2-5p
198		hsa-miR-499a-5p
199		hsa-miR-501-5p
200		hsa-miR-502-5p
201		hsa-miR-503-5p
202		hsa-miR-503-3p
203		hsa-miR-504-5p
204		hsa-miR-504-3p
205		hsa-miR-508-5p
206		hsa-miR-532-3p
207		hsa-miR-455-3p
208		hsa-miR-376a-2-5p
209		hsa-miR-376a-2-5p
210		hsa-miR-92b-5p
211		hsa-miR-92b-3p
212		hsa-miR-557
213		hsa-miR-564
214		hsa-miR-574-5p
215		hsa-miR-574-3p
216		hsa-miR-579-5p
217		hsa-miR-584-3p
218		hsa-miR-584-3p
219		hsa-miR-585-5p
220		hsa-miR-548b-3p
221		hsa-miR-588
222		hsa-miR-550a-5p
223		hsa-miR-593-3p
224		hsa-miR-595
225		hsa-miR-595
226		hsa-miR-596
227		hsa-miR-597-3p
228		hsa-miR-598-3p
229		hsa-miR-600
230		hsa-miR-601
231		hsa-miR-601
232		hsa-miR-603
233		hsa-miR-608
234		hsa-miR-609
235		hsa-miR-610
236		hsa-miR-611
237		hsa-miR-611
238		hsa-miR-611
239		hsa-miR-614
240		hsa-miR-615-5p
241		hsa-miR-615-3p
242		hsa-miR-615-3p
243		hsa-miR-548c-5p
244		hsa-miR-619-5p
245		hsa-miR-619-3p
246		hsa-miR-623
247		hsa-miR-624-5p
248		hsa-miR-625-3p
249		hsa-miR-625-3p
250		hsa-miR-631
251		hsa-miR-33b-3p
252		hsa-miR-632
253		hsa-miR-638
254		hsa-miR-642a-5p
255		hsa-miR-643
256		hsa-miR-647
257		hsa-miR-647
258		hsa-miR-652-5p
259		hsa-miR-661
260		hsa-miR-662
261		hsa-miR-663a
262		hsa-miR-449b-5p
263		hsa-miR-449b-3p
264		hsa-miR-654-5p
265		hsa-miR-654-5p
266		hsa-miR-654-3p
267		hsa-miR-549a-5p
268		hsa-miR-1264
269		hsa-miR-668-5p
270		hsa-miR-550a-3-5p
271		hsa-miR-550a-3-5p
272		hsa-miR-767-5p
273		hsa-miR-1224-3p
274		hsa-miR-320b
275		hsa-miR-320b
276		hsa-miR-320c
277		hsa-miR-1296-5p
278		hsa-miR-1301-3p
279		hsa-miR-1185-2-3p
280		hsa-miR-761
281		hsa-miR-764
282		hsa-miR-770-5p
283		hsa-miR-675-5p
284		hsa-miR-675-3p
285		hsa-miR-874-3p
286		hsa-miR-874-3p
287		hsa-miR-890
288		hsa-miR-888-3p
289		hsa-miR-892b
290		hsa-miR-889-5p
291		hsa-miR-708-3p
292		hsa-miR-744-5p
293		hsa-miR-877-5p
294		hsa-miR-877-3p
295		hsa-miR-543
296		hsa-miR-760
297		hsa-miR-920
298		hsa-miR-509-3-5p
299		hsa-miR-933
300		hsa-miR-937-3p
301		hsa-miR-939-3p
302		hsa-miR-940
303		hsa-miR-941
304		hsa-miR-941
305		hsa-miR-942-5p
306		hsa-miR-943
307		hsa-miR-1178-5p
308		hsa-miR-1180-3p
309		hsa-miR-1227-3p
310		hsa-miR-1236-3p
311		hsa-miR-1237-3p
312		hsa-miR-1238-3p
313		hsa-miR-1200
314		hsa-miR-1285-5p
315		hsa-miR-1286
316		hsa-miR-1287-3p
317		hsa-miR-1289
318		hsa-miR-1293
319		hsa-miR-1294
320		hsa-miR-1295a
321		hsa-miR-1299
322		hsa-miR-1304-3p
323		hsa-miR-1250-3p
324		hsa-miR-1250-3p
325		hsa-miR-1251-5p
326		hsa-miR-1255a
327		hsa-miR-548g-5p
328		hsa-miR-1261
329		hsa-miR-1261
330		hsa-miR-1263
331		hsa-miR-1266-5p
332		hsa-miR-1266-3p
333		hsa-miR-1266-3p
334		hsa-miR-1266-3p
335		hsa-miR-1268a
336		hsa-miR-1275
337		hsa-miR-548i
338		hsa-miR-1281
339		hsa-miR-1307-3p
340		hsa-miR-513b-3p
341		hsa-miR-1324
342		hsa-miR-1469
343		hsa-miR-1469
344		hsa-miR-1538
345		hsa-miR-1827
346		hsa-miR-1909-5p
347		hsa-miR-1909-5p
348		hsa-miR-1911-5p
349		hsa-miR-1911-3p
350		hsa-miR-1913
351		hsa-miR-2110
352		hsa-miR-2110
353		hsa-miR-2114-5p
354		hsa-miR-2114-5p
355		hsa-miR-2117
356		hsa-miR-2276-5p
357		hsa-miR-2276-3p
358		hsa-miR-2682-3p
359		hsa-miR-2682-3p
360		hsa-miR-711
361		hsa-miR-2861
362		hsa-miR-3120-5p
363		hsa-miR-3124-3p
364		hsa-miR-3127-5p
365		hsa-miR-3127-3p
366		hsa-miR-3130-5p
367		hsa-miR-3130-5p
368		hsa-miR-3130-5p
369		hsa-miR-3132
370		hsa-miR-3132
371		hsa-miR-3135a
372		hsa-miR-3137
373		hsa-miR-3139
374		hsa-miR-3141
375		hsa-miR-3141
376		hsa-miR-3144-5p
377		hsa-miR-3147
378		hsa-miR-3147
379		hsa-miR-3150a-3p
380		hsa-miR-3152-5p
381		hsa-miR-3152-3p
382		hsa-miR-3074-3p
383		hsa-miR-3154
384		hsa-miR-3154
385		hsa-miR-3155a
386		hsa-miR-3155a
387		hsa-miR-3156-5p
388		hsa-miR-3156-5p
389		hsa-miR-3156-3p
390		hsa-miR-3157-5p
391		hsa-miR-3157-5p
392		hsa-miR-3158-3p
393		hsa-miR-3160-5p
394		hsa-miR-3161
395		hsa-miR-3162-5p
396		hsa-miR-3162-5p
397		hsa-miR-3169
398		hsa-miR-3173-5p
399		hsa-miR-323b-5p
400		hsa-miR-323b-5p
401		hsa-miR-3176
402		hsa-miR-3177-3p
403		hsa-miR-3178
404		hsa-miR-3180-5p
405		hsa-miR-3065-3p
406		hsa-miR-3186-5p
407		hsa-miR-3186-5p
408		hsa-miR-3187-5p
409		hsa-miR-3188
410		hsa-miR-3189-3p
411		hsa-miR-3189-3p
412		hsa-miR-3190-5p
413		hsa-miR-3190-3p
414		hsa-miR-3191-5p
415		hsa-miR-3191-5p
416		hsa-miR-3191-3p
417		hsa-miR-3192-3p
418		hsa-miR-3194-5p
419		hsa-miR-3196
420		hsa-miR-548x-5p
421		hsa-miR-3199
422		hsa-miR-3199
423		hsa-miR-3200-5p
424		hsa-miR-514b-5p
425		hsa-miR-3202
426		hsa-miR-4296
427		hsa-miR-4297
428		hsa-miR-4294
429		hsa-miR-4298
430		hsa-miR-4298
431		hsa-miR-4313
432		hsa-miR-4258
433		hsa-miR-4259
434		hsa-miR-4260
435		hsa-miR-4253
436		hsa-miR-4254
437		hsa-miR-4252
438		hsa-miR-4325
439		hsa-miR-4268
440		hsa-miR-4263
441		hsa-miR-4277
442		hsa-miR-4278
443		hsa-miR-4284
444		hsa-miR-4286
445		hsa-miR-4292
446		hsa-miR-4289
447		hsa-miR-4289
448		hsa-miR-4290
449		hsa-miR-4330
450		hsa-miR-3605-5p
451		hsa-miR-3605-3p
452		hsa-miR-3615
453		hsa-miR-3616-3p
454		hsa-miR-3619-5p
455		hsa-miR-3620-5p
456		hsa-miR-3620-3p
457		hsa-miR-3622a-3p
458		hsa-miR-3648
459		hsa-miR-3652
460		hsa-miR-3655
461		hsa-miR-3660
462		hsa-miR-3661
463		hsa-miR-3663-5p
464		hsa-miR-3663-3p
465		hsa-miR-3663-3p
466		hsa-miR-3667-3p
467		hsa-miR-3667-3p
468		hsa-miR-3670
469		hsa-miR-3679-3p
470		hsa-miR-3690
471		hsa-miR-3692-5p
472		hsa-miR-3180
473		hsa-miR-3908
474		hsa-miR-3909
475		hsa-miR-3150b-5p
476		hsa-miR-3927-3p
477		hsa-miR-3929
478		hsa-miR-3935
479		hsa-miR-3936
480		hsa-miR-3939
481		hsa-miR-3940-5p
482		hsa-miR-3940-3p
483		hsa-miR-3940-3p
484		hsa-miR-550b-2-5p
485		hsa-miR-548o-5p
486		hsa-miR-1268b
487		hsa-miR-378e
488		hsa-miR-378g
489		hsa-miR-4428
490		hsa-miR-4433a-3p
491		hsa-miR-4435
492		hsa-miR-4439
493		hsa-miR-4440
494		hsa-miR-4445-5p
495		hsa-miR-4446-3p
496		hsa-miR-4449
497		hsa-miR-4452
498		hsa-miR-4455
499		hsa-miR-4462
500		hsa-miR-548aj-5p
501		hsa-miR-4466
502		hsa-miR-4469
503		hsa-miR-4474-3p
504		hsa-miR-4477a
505		hsa-miR-3689f
506		hsa-miR-4479
507		hsa-miR-4481
508		hsa-miR-4482-5p
509		hsa-miR-4487
510		hsa-miR-4488
511		hsa-miR-4489
512		hsa-miR-4489
513		hsa-miR-4491
514		hsa-miR-4493
515		hsa-miR-4494
516		hsa-miR-4502
517		hsa-miR-2392
518		hsa-miR-4507
519		hsa-miR-4507
520		hsa-miR-4508
521		hsa-miR-4510
522		hsa-miR-4515
523		hsa-miR-4520-5p
524		hsa-miR-4522
525		hsa-miR-4533
526		hsa-miR-548am-5p
527		hsa-miR-4536-5p
528		hsa-miR-3960
529		hsa-miR-3972
530		hsa-miR-3975
531		hsa-miR-4632-5p
532		hsa-miR-4632-3p
533		hsa-miR-4633-5p
534		hsa-miR-4633-3p
535		hsa-miR-4634
536		hsa-miR-4638-5p
537		hsa-miR-4638-5p
538		hsa-miR-4639-5p
539		hsa-miR-4640-5p
540		hsa-miR-4640-3p
541		hsa-miR-4641
542		hsa-miR-4646-5p
543		hsa-miR-4646-3p
544		hsa-miR-4646-3p
545		hsa-miR-4650-5p
546		hsa-miR-4650-3p
547		hsa-miR-4651
548		hsa-miR-4651
549		hsa-miR-4651
550		hsa-miR-4651
551		hsa-miR-4652-3p
552		hsa-miR-4653-5p
553		hsa-miR-4653-3p
554		hsa-miR-4653-3p
555		hsa-miR-4655-5p
556		hsa-miR-4655-3p
557		hsa-miR-4656
558		hsa-miR-4657
559		hsa-miR-4659a-5p
560		hsa-miR-4660
561		hsa-miR-4664-5p
562		hsa-miR-4664-5p
563		hsa-miR-4664-3p
564		hsa-miR-4664-3p
565		hsa-miR-4665-5p
566		hsa-miR-4667-3p
567		hsa-miR-4674
568		hsa-miR-4675
569		hsa-miR-4676-3p
570		hsa-miR-4677-5p
571		hsa-miR-4677-3p
572		hsa-miR-4684-3p
573		hsa-miR-4685-3p
574		hsa-miR-4685-3p
575		hsa-miR-4687-5p
576		hsa-miR-4687-3p
577		hsa-miR-4687-3p
578		hsa-miR-1343-5p
579		hsa-miR-4690-5p
580		hsa-miR-4691-5p
581		hsa-miR-4694-5p
582		hsa-miR-4695-5p
583		hsa-miR-4695-3p
584		hsa-miR-4695-3p
585		hsa-miR-4696
586		hsa-miR-4697-5p
587		hsa-miR-4701-3p
588		hsa-miR-4707-3p
589		hsa-miR-4708-5p
590		hsa-miR-203b-5p
591		hsa-miR-203b-3p
592		hsa-miR-4710
593		hsa-miR-4712-5p
594		hsa-miR-4713-3p
595		hsa-miR-4714-5p
596		hsa-miR-4714-5p
597		hsa-miR-4714-5p
598		hsa-miR-4716-3p
599		hsa-miR-3529-5p
600		hsa-miR-3529-5p
601		hsa-miR-4717-5p
602		hsa-miR-4717-3p
603		hsa-miR-4717-3p
604		hsa-miR-4717-3p
605		hsa-miR-4718
606		hsa-miR-4721
607		hsa-miR-4722-3p
608		hsa-miR-4723-3p
609		hsa-miR-4725-3p
610		hsa-miR-4726-3p
611		hsa-miR-4727-5p
612		hsa-miR-4728-3p
613		hsa-miR-4731-5p
614		hsa-miR-4732-3p
615		hsa-miR-4732-3p
616		hsa-miR-4732-3p
617		hsa-miR-3064-5p
618		hsa-miR-3064-5p
619		hsa-miR-4739
620		hsa-miR-4741
621		hsa-miR-4742-5p
622		hsa-miR-4743-3p
623		hsa-miR-4745-3p
624		hsa-miR-4746-3p
625		hsa-miR-4747-3p
626		hsa-miR-4750-5p
627		hsa-miR-371b-3p
628		hsa-miR-4754
629		hsa-miR-4755-5p
630		hsa-miR-4755-5p
631		hsa-miR-499b-5p
632		hsa-miR-499b-5p
633		hsa-miR-4756-5p
634		hsa-miR-4756-5p
635		hsa-miR-4756-3p
636		hsa-miR-4757-5p
637		hsa-miR-4758-5p
638		hsa-miR-4758-3p
639		hsa-miR-4762-3p
640		hsa-miR-4767
641		hsa-miR-4769-3p
642		hsa-miR-4773
643		hsa-miR-4774-5p
644		hsa-miR-4774-5p
645		hsa-miR-4774-3p
646		hsa-miR-4436b-3p
647		hsa-miR-4784
648		hsa-miR-2467-3p
649		hsa-miR-4786-3p
650		hsa-miR-4789-3p
651		hsa-miR-4793-5p
652		hsa-miR-4793-3p
653		hsa-miR-4794
654		hsa-miR-4799-3p
655		hsa-miR-4800-5p
656		hsa-miR-4800-3p
657		hsa-miR-4804-3p
658		hsa-miR-4999-5p
659		hsa-miR-5000-5p
660		hsa-miR-5001-3p
661		hsa-miR-5002-5p
662		hsa-miR-5003-3p
663		hsa-miR-5006-5p
664		hsa-miR-5006-3p
665		hsa-miR-5007-5p
666		hsa-miR-5008-5p
667		hsa-miR-5047
668		hsa-miR-5087
669		hsa-miR-5090
670		hsa-miR-5094
671		hsa-miR-5187-5p
672		hsa-miR-5192
673		hsa-miR-5193
674		hsa-miR-5196-3p
675		hsa-miR-5196-3p
676		hsa-miR-5572
677		hsa-miR-5572
678		hsa-miR-548as-5p
679		hsa-miR-5579-3p
680		hsa-miR-664b-3p
681		hsa-miR-5581-3p
682		hsa-miR-5584-3p
683		hsa-miR-1295b-3p
684		hsa-miR-5588-3p
685		hsa-miR-5589-5p
686		hsa-miR-5589-3p
687		hsa-miR-5590-3p
688		hsa-miR-5689
689		hsa-miR-5692a
690		hsa-miR-4666b
691		hsa-miR-5697
692		hsa-miR-5698
693		hsa-miR-5699-5p
694		hsa-miR-5704
695		hsa-miR-5705
696		hsa-miR-5739
697		hsa-miR-5787
698		hsa-miR-1199-3p
699		hsa-miR-6071
700		hsa-miR-6073
701		hsa-miR-6073
702		hsa-miR-6073
703		hsa-miR-6075
704		hsa-miR-6076
705		hsa-miR-6078
706		hsa-miR-6078
707		hsa-miR-6085
708		hsa-miR-6086
709		hsa-miR-6088
710		hsa-miR-6090
711		hsa-miR-6130
712		hsa-miR-6130
713		hsa-miR-6131
714		hsa-miR-6134
715		hsa-miR-6500-5p
716		hsa-miR-6500-5p
717		hsa-miR-6500-3p
718		hsa-miR-6501-5p
719		hsa-miR-6501-5p
720		hsa-miR-6502-5p
721		hsa-miR-6503-5p
722		hsa-miR-6504-5p
723		hsa-miR-6504-3p
724		hsa-miR-6504-3p
725		hsa-miR-6507-3p
726		hsa-miR-6509-5p
727		hsa-miR-6509-5p
728		hsa-miR-6510-5p
729		hsa-miR-6511a-3p
730		hsa-miR-6512-3p
731		hsa-miR-6513-3p
732		hsa-miR-6514-5p
733		hsa-miR-6514-3p
734		hsa-miR-6515-5p
735		hsa-miR-6515-5p
736		hsa-miR-6515-3p
737		hsa-miR-6715a-3p
738		hsa-miR-6715b-3p
739		hsa-miR-6716-5p
740		hsa-miR-6716-3p
741		hsa-miR-6717-5p
742		hsa-miR-6511b-5p
743		hsa-miR-6511b-3p
744		hsa-miR-6718-5p
745		hsa-miR-6718-5p
746		hsa-miR-6718-5p
747		hsa-miR-6720-5p
748		hsa-miR-6720-3p
749		hsa-miR-6721-5p
750		hsa-miR-892c-3p
751		hsa-miR-6726-5p
752		hsa-miR-6726-3p
753		hsa-miR-6726-3p
754		hsa-miR-6727-3p
755		hsa-miR-6728-3p
756		hsa-miR-6730-5p
757		hsa-miR-6730-3p
758		hsa-miR-6731-3p
759		hsa-miR-6732-5p
760		hsa-miR-6732-5p
761		hsa-miR-6732-3p
762		hsa-miR-6734-5p
763		hsa-miR-6734-5p
764		hsa-miR-6735-5p
765		hsa-miR-6736-5p
766		hsa-miR-6737-3p
767		hsa-miR-6741-3p
768		hsa-miR-6741-3p
769		hsa-miR-6742-5p
770		hsa-miR-6742-5p
771		hsa-miR-6744-5p
772		hsa-miR-6746-5p
773		hsa-miR-6746-5p
774		hsa-miR-6746-3p
775		hsa-miR-6747-5p
776		hsa-miR-6747-3p
777		hsa-miR-6748-3p
778		hsa-miR-6749-5p
779		hsa-miR-6749-5p
780		hsa-miR-6750-5p
781		hsa-miR-6750-3p
782		hsa-miR-6751-5p
783		hsa-miR-6753-3p
784		hsa-miR-6754-5p
785		hsa-miR-6756-3p
786		hsa-miR-6757-5p
787		hsa-miR-6758-5p
788		hsa-miR-6758-5p
789		hsa-miR-6758-3p
790		hsa-miR-6759-5p
791		hsa-miR-6760-5p
792		hsa-miR-6760-3p
793		hsa-miR-6760-3p
794		hsa-miR-6761-5p
795		hsa-miR-6763-5p
796		hsa-miR-6763-3p
797		hsa-miR-6763-3p
798		hsa-miR-6764-3p
799		hsa-miR-6766-3p
800		hsa-miR-6766-3p
801		hsa-miR-6770-5p
802		hsa-miR-6770-3p
803		hsa-miR-6770-3p
804		hsa-miR-6771-5p
805		hsa-miR-6773-3p
806		hsa-miR-6773-3p
807		hsa-miR-6774-3p
808		hsa-miR-6775-3p
809		hsa-miR-6776-5p
810		hsa-miR-6776-3p
811		hsa-miR-6777-3p
812		hsa-miR-6778-5p
813		hsa-miR-6782-5p
814		hsa-miR-6782-3p
815		hsa-miR-6783-3p
816		hsa-miR-6785-5p
817		hsa-miR-6788-5p
818		hsa-miR-6788-5p
819		hsa-miR-6788-5p
820		hsa-miR-6788-5p
821		hsa-miR-6789-3p
822		hsa-miR-6789-3p
823		hsa-miR-6790-5p
824		hsa-miR-6791-3p
825		hsa-miR-6793-5p
826		hsa-miR-6793-3p
827		hsa-miR-6793-3p
828		hsa-miR-6794-5p
829		hsa-miR-6794-3p
830		hsa-miR-6795-5p
831		hsa-miR-6795-5p
832		hsa-miR-6795-5p
833		hsa-miR-6795-3p
834		hsa-miR-6796-5p
835		hsa-miR-6796-3p
836		hsa-miR-6799-3p
837		hsa-miR-6800-3p
838		hsa-miR-6801-3p
839		hsa-miR-6802-3p
840		hsa-miR-6803-5p
841		hsa-miR-6805-3p
842		hsa-miR-6806-5p
843		hsa-miR-6806-3p
844		hsa-miR-6807-3p
845		hsa-miR-6808-5p
846		hsa-miR-6809-3p
847		hsa-miR-6811-5p
848		hsa-miR-6811-5p
849		hsa-miR-6811-3p
850		hsa-miR-6812-5p
851		hsa-miR-6812-5p
852		hsa-miR-6813-5p
853		hsa-miR-6814-5p
854		hsa-miR-6814-3p
855		hsa-miR-6815-5p
856		hsa-miR-6816-5p
857		hsa-miR-6817-3p
858		hsa-miR-6817-3p
859		hsa-miR-6820-3p
860		hsa-miR-6820-3p
861		hsa-miR-6821-3p
862		hsa-miR-6823-5p
863		hsa-miR-6824-5p
864		hsa-miR-6825-5p
865		hsa-miR-6827-5p
866		hsa-miR-6827-3p
867		hsa-miR-6827-3p
868		hsa-miR-6828-3p
869		hsa-miR-6829-5p
870		hsa-miR-6830-5p
871		hsa-miR-6831-5p
872		hsa-miR-6832-3p
873		hsa-miR-6833-5p
874		hsa-miR-6833-3p
875		hsa-miR-6833-3p
876		hsa-miR-6834-5p
877		hsa-miR-6834-5p
878		hsa-miR-6834-3p
879		hsa-miR-6780b-5p
880		hsa-miR-6837-3p
881		hsa-miR-6838-5p
882		hsa-miR-6841-3p
883		hsa-miR-6842-5p
884		hsa-miR-6843-3p
885		hsa-miR-6846-5p
886		hsa-miR-6847-5p
887		hsa-miR-6849-3p
888		hsa-miR-6849-3p
889		hsa-miR-6850-5p
890		hsa-miR-6851-5p
891		hsa-miR-6852-5p
892		hsa-miR-6854-5p
893		hsa-miR-6854-3p
894		hsa-miR-6855-5p
895		hsa-miR-6856-5p
896		hsa-miR-6769b-5p
897		hsa-miR-6769b-5p
898		hsa-miR-6860
899		hsa-miR-6862-5p
900		hsa-miR-6862-3p
901		hsa-miR-6863
902		hsa-miR-6865-5p
903		hsa-miR-6865-5p
904		hsa-miR-6865-3p
905		hsa-miR-6865-3p
906		hsa-miR-6865-3p
907		hsa-miR-6867-5p
908		hsa-miR-6868-5p
909		hsa-miR-6868-3p
910		hsa-miR-6868-3p
911		hsa-miR-6868-3p
912		hsa-miR-6869-5p
913		hsa-miR-6870-5p
914		hsa-miR-6870-5p
915		hsa-miR-6870-3p
916		hsa-miR-6870-3p
917		hsa-miR-6870-3p
918		hsa-miR-6871-3p
919		hsa-miR-6872-5p
920		hsa-miR-6872-5p
921		hsa-miR-6872-3p
922		hsa-miR-6873-5p
923		hsa-miR-6875-5p
924		hsa-miR-6875-5p
925		hsa-miR-6875-3p
926		hsa-miR-6877-3p
927		hsa-miR-6878-3p
928		hsa-miR-6879-5p
929		hsa-miR-6880-5p
930		hsa-miR-6880-5p
931		hsa-miR-6881-3p
932		hsa-miR-6882-3p
933		hsa-miR-6883-5p
934		hsa-miR-6883-3p
935		hsa-miR-6885-5p
936		hsa-miR-6886-3p
937		hsa-miR-6887-5p
938		hsa-miR-6887-3p
939		hsa-miR-6888-5p
940		hsa-miR-6889-3p
941		hsa-miR-6889-3p
942		hsa-miR-6890-5p
943		hsa-miR-6890-5p
944		hsa-miR-6892-3p
945		hsa-miR-6893-5p
946		hsa-miR-6894-3p
947		hsa-miR-6895-3p
948		hsa-miR-7106-5p
949		hsa-miR-7106-5p
950		hsa-miR-7107-5p
951		hsa-miR-7107-3p
952		hsa-miR-7108-3p
953		hsa-miR-7108-3p
954		hsa-miR-7109-5p
955		hsa-miR-7109-3p
956		hsa-miR-7110-5p
957		hsa-miR-7110-5p
958		hsa-miR-7111-5p
959		hsa-miR-7112-3p
960		hsa-miR-7114-5p
961		hsa-miR-7114-3p
962		hsa-miR-7150
963		hsa-miR-7152-3p
964		hsa-miR-7152-3p
965		hsa-miR-7155-3p
966		hsa-miR-7156-3p
967		hsa-miR-7161-3p
968		hsa-miR-7160-5p
969		hsa-miR-7162-5p
970		hsa-miR-7702
971		hsa-miR-7703
972		hsa-miR-7704
973		hsa-miR-7706
974		hsa-miR-7843-3p
975		hsa-miR-7843-3p
976		hsa-miR-7843-3p
977		hsa-miR-4433b-5p
978		hsa-miR-1273h-5p
979		hsa-miR-7845-5p
980		hsa-miR-7846-3p
981		hsa-miR-7847-3p
982		hsa-miR-7847-3p
983		hsa-miR-7849-3p
984		hsa-miR-7850-5p
985		hsa-miR-7854-3p
986		hsa-miR-7974
987		hsa-miR-7975
988		hsa-miR-7976
989		hsa-miR-7976
990		hsa-miR-7976
991		hsa-miR-8052
992		hsa-miR-8058
993		hsa-miR-8071
994		hsa-miR-8071
995		hsa-miR-8073
996		hsa-miR-8077
997		hsa-miR-8089
998		hsa-miR-8485
999		hsa-miR-548bb-5p
1000		hsa-miR-9903
1001		hsa-miR-1843
1002		hsa-miR-1843
1003		hsa-miR-10226
1004		hsa-miR-10392-3p
1005		hsa-miR-10394-5p
1006		hsa-miR-10394-3p
1007		hsa-miR-10397-5p
1008		hsa-miR-10398-5p
1009		hsa-miR-10398-5p
1010		hsa-miR-10399-5p
1011		hsa-miR-10400-5p
1012		hsa-miR-10401-5p
1013		hsa-miR-10401-3p
1014		hsa-miR-10401-3p
1015		hsa-miR-10396b-3p
1016		hsa-miR-10522-5p
1017		hsa-miR-10524-5p
1018		hsa-miR-3059-5p
1019		hsa-miR-3085-3p
1020		hsa-miR-6529-5p
1021		hsa-miR-9851-3p
1022		hsa-miR-12114
1023		hsa-miR-12128
1024		hsa-miR-12128
1025		hsa-miR-12129
1026		hsa-miR-12131

miRNA = microRNA.

## 4. Discussion

As far as we know, there were relatively few studies on the related mechanism of ferroptosis in the occurrence of AAA. We reported for the first time that circRNA-miRNA-mRNA axis was constructed on the premise of ferroptosis-related genes to analyze the pathogenesis of AAA.

Our analysis of microarray data first revealed that the ferroptosis-associated gene AKR1C1 had low expression in the AAA group compared to the normal group and may play a protective role. Four proteins, AKR1C1, AKR1C2, AKR1C3 and AKR1C4, members of the AKR1C subfamily, were identified by the Human Genome Project.^[27]^ AKR1C subtype has more than 86% identical amino acid sequences, especially AKR1C1 and AKR1C2, which have 97% homologous sequences.^[28,29]^ AKR1C1 was involved in the metabolism of many endogenous and exogenous compounds in the body, including steroids, fat, glucocorticoids and prostaglandins.^[30]^ AKR1C1, as the main 20-keto steroid reductase in human body, can metabolize progesterone into 20α -hydroxyprogesterone and participate in maintaining circulating progesterone levels.^[31]^ Mutation of AKR1C1 was an important cause of sexual dysplasia.^[32]^ In addition, studies have reported that the high expression of AKR1C1 was closely related to the good prognosis of breast cancer and neuroblastoma.^[33,34]^

In general, miRNAs are small non-coding RNAs, 21-25 nucleotides in length, that inhibit translation or induce degradation of target mRNAs at the post-transcriptional level by binding to the 3’ untranslated region of the gene.^[35,36]^ We predicted the potential binding sites between AKR1C1 and miRNA-543 by TargetScan and miRWalk database. It has been reported that miRNA-543 downregulated SIRT1/AMPK/ NF-Kappab signaling pathway to reduce inflammatory response and myocardial injury in children with viral myocarditis.^[37]^ MiRNA-543 mediated COL4A1 expression in exosomes derived from human mesenchymal stem cells to promote angiogenesis of cardiac microvascular endothelial cells after myocardial infarction.^[38]^ The above studies suggest that miRNA-543 may be involved in the formation of cardiovascular diseases, but no relevant reports have been reported in AAA.

CircRNA is a single-stranded RNA transcript without a 5’ cap or 3’ polyadenylate tail, and is a covalent closed-loop structure generated by reverse splicing of pre-mRNA.^[39]^ Some circRNAs in eukaryotes have been identified as having cell- or tissue-specific expression patterns and high stability and evolutionary conservatism.^[40]^ Initial studies have shown that circRNA can mediate miRNA function and regulate the transcription process.^[41]^ Recent studies found that circRNA mitigated the targeted inhibition of miRNA on downstream mRNA by binding miRNA response element, which was also known as the ceRNA regulatory mechanism.^[42,43]^ Has_circ_0005073/miRNA-543/AKR1C1 axis mediated AAA lesions was precisely the above mechanism of action.

In addition, previous studies have shown that AAA aortic tissue iron levels are significantly increased, and the inflammatory response induced by oxidative stress and iron overload aggravates the progression of AAA.^[16]^ Recent studies have found that ganglioside GM3 supplementation significantly inhibits lipid peroxidation, reduces iron deposition in aortic VSMC, and significantly reduces the incidence of AAA.^[44]^ Another study demonstrated the important role of neutrophil extracellular trap-induced ferroptosis of VSMC in AAA formation, and found that mesenchymal stem cell-derived extracellular vesicles may play a therapeutic role in AAA through their immunomodulatory and regenerative capacity.^[45]^ The conclusions reached by the above studies are similar to those of the present study, and all confirm the important role of ferroptosis-related proteins in the occurrence and development of AAA. Importantly, to our knowledge, we are the first to propose a potential relationship between the ferroptosis gene AKR1C1 and AAA.

However, there are some limitations to our study. First, AAA-related clinical information is missing in public databases. Secondly, the regulatory relationship among circRNA, miRNA and mRNA has not been confirmed by double luciferase reporter gene analysis, pull down, gene overexpression or gene knockout. In addition, further in vitro and in vivo experiments should be performed to confirm the present results. Finally, we will search for potential drugs that may be regulated through Drugbank database in the future, while verifying the potential of miRNA-543 as a serological marker in clinical patients.

## 5. Conclusion

We revealed the potential regulatory network of AAA by constructing the circRNA-miRNA-mRNA axis that may be involved in the occurrence and development of AAA. At the same time, the above key factors can be used as the serum diagnostic markers of AAA and the development targets of drug targeted intervention.

## Author contributions

**Conceptualization:** Xuehua Huang.

**Data curation:** Xuehua Huang.

**Formal analysis:** Xuehua Huang.

**Investigation:** Xuehua Huang.

**Methodology:** Huanhuan Deng.

**Resources:** Huanhuan Deng.

**Supervision:** Huanhuan Deng.

**Visualization:** Huanhuan Deng.

**Writing – original draft:** Xuehua Huang.

**Writing – review & editing:** Huanhuan Deng.
